# High level of BRD4 promotes non-small cell lung cancer progression

**DOI:** 10.18632/oncotarget.7068

**Published:** 2016-01-29

**Authors:** Yun-Fei Liao, Yong-Bing Wu, Xiang Long, Shu-Qiang Zhu, Chun Jin, Jian-Jun Xu, Jian-Yong Ding

**Affiliations:** ^1^ Department of Thoracic Surgery, The Affiliated Zhongshan Hospital of Fudan University, Shanghai 200032, P. R. China; ^2^ Department of Cardiothoracic Surgery, The Second Affiliated Hospital of Nanchang University, Nanchang 330000, P. R. China

**Keywords:** non-small cell lung cancer, bromodomain containing protein 4, proliferation, invasion, apoptosis

## Abstract

Bromodomain containing protein 4 (BRD4), a member of the bromodomain and extra terminal domain (BET) protein family, has been shown to play important roles in tumor progression. However, its role in non-small cell lung cancer (NSCLC) is still largely unknown. Here, we found that BRD4 expression was significantly upregulated in NSCLC tissues and NSCLC cell lines with higher invasion and metastasis potentials. Suppression of BRD4 expression in NSCLC cell lines impaired cell invasion, inhibited cell proliferation, and accelerated cell apoptosis. Clinically, we observed that the BRD4 level was significantly related to histological type, lymph node metastasis, tumor stage and differentiation. More importantly, high level of BRD4 was closely correlated with the poor prognosis of NSCLC patients. Therefore, our study suggests that BRD4 is one of the major contributors to the invasion-prone phenotype of NSCLC, and a potential therapeutic target of NSCLC.

## INTRODUCTION

Lung cancer is the first leading cause of cancer-related deaths worldwide [1]. The non-small cell lung cancer (NSCLC), which accounts for about 85% of lung cancers, is the most common type of lung cancers. To date, surgical intervention is still the preferred form of therapy of NSCLC, but its high recurrent rate after curative lobectomy resection and relatively insensitive to adjuvant chemotherapy preclude surgical treatment from being curative in most NSCLC patients [2]. Therefore, looking for a new biomarker relevant to this disease is of paramount importance in order to develop new preventive, diagnostic, and therapeutic options.

Bromodomain containing protein 4 (BRD4) belongs to the protein of the bromodomain and extraterminal domain (BET) family [3]. Similar to its BET family members, BRD4 includes two bromodomains that recognize acetylated lysine residues [4]. BRD4 is the best-studied member of the BET family and it plays an important role in various biological processes by means of its two bromodomains (BRDs). It links cell cycle and transcription, bookmarking active genes during mitosis and serving as a scaffold for transcription factors. Moreover, BRD4 is a nuclear protein that binds to acetylated histone 3 (H3) and histone 4 (H4) tails and plays an important role in maintaining chromatin architecture and controls transcription elongation through phosphorylation of RNA polymerase II [7–11]. More interestingly, recent studies have also showed that BRD4 activation may also predict the overall survival of patients with several tumors, such as melanoma, hepatocellular carcinoma, multiple myeloma, Burkitt's lymphoma, acute myeloid leukemia and breast cancer [12–17].

Although BRD4 has been regarded as a potential therapeutic target for many diseases including some cancers, it remains elusive in NSCLC. Therefore, this study aimed to investigate whether BRD4 is one of the major contributors to the invasion-prone phenotype of NSCLC, and define the protein as a suitable candidate for therapeutic agents of NSCLC patients.

## RESULTS

### BRD4 highly expresses in NSCLC tissues and NSCLC cell lines with higher invasion and metastasis potentials

**Figure 1:**
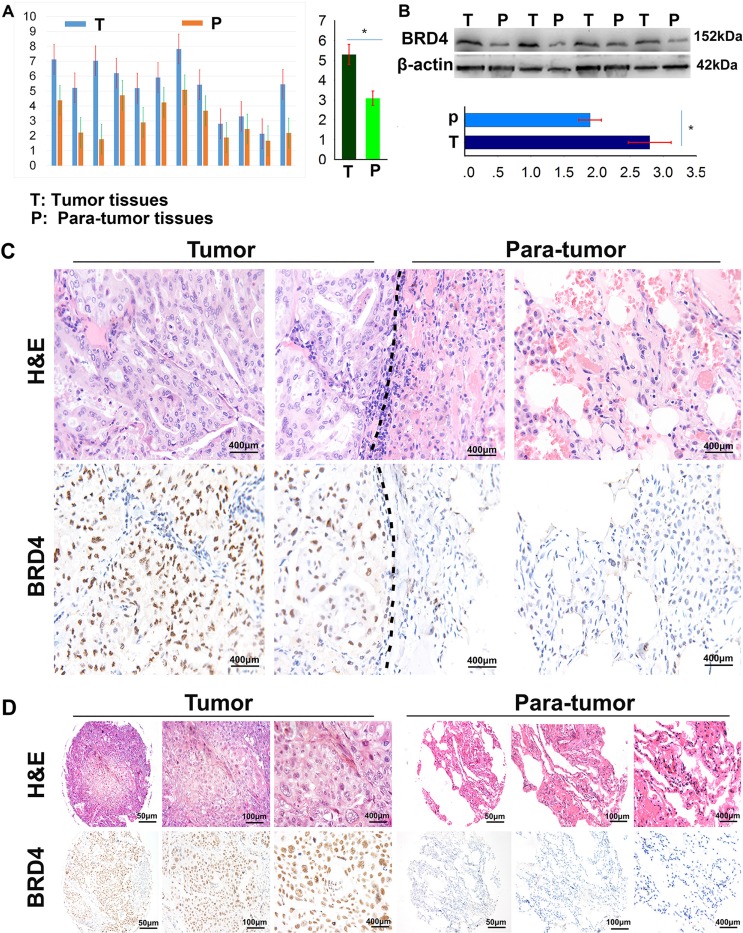
BRD4 expresses in NSCLC tissues and cell lines (**A**) The BRD4 expression in 12 NSCLC tissues and their matched adjacent lung tissues was detected by qRT-PCR (*p* < 0.05); (**B**) The BRD4 expression in 12 NSCLC tissues and their matched adjacent lung tissues was detected by western blot (*p* < 0.05); (**C**) The BRD4 expression in NSCLC tissues and their matched adjacent lung tissues was detected by immunohistochemistry on formalin-fixed sections. (**D**) Representative pictures of immunohistochemical staining present the expression of BRD4 in NSCLC and their corresponding adjacent normal lung tissues in tissue microarrays.

To specifically address the association of BRD4 expression in NSCLC tissues with the disease prognosis, we firstly detected the BRD4 expression in NSCLC and their corresponding adjacent normal lung tissues by real time-quantitative polymerase chain reaction (qRT-PCR) and western blot. As shown in Figure 1A and 1B, the BRD4 expression was much higher in NSCLC tissues than their corresponding adjacent normal lung tissues in mRNA (5.30 ± 0.51 *vs.* 3.10 ± 0.36) and protein (2.80 ± 0.32 *vs.* 1.90 ± 0.17) level. By immunohistochemistry, we found that the BRD4 staining was localized to the nucleus, and the expression of BRD4 in NSCLC tissues was evidently stronger than that in their corresponding adjacent normal lung tissues (Figure 1C), and the difference was significant (Figure 1D).

**Figure 2:**
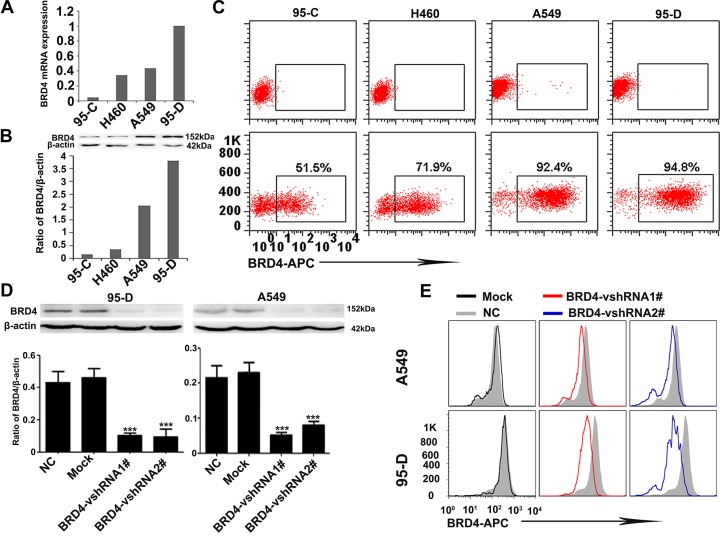
BRD4 expression is positively associated with the invasion and metastasis potentials of NSCLC cells and interference of BRD4 in 95-D and A549 cells (**A**) BRD4 expression in several NSCLC cell lines at mRNA level. (**B**) Western blotting shows that BRD4 highly expresses in A549 and 95-D cell lines. (**C**) The flow cytometry presents the BRD4 expression in NSCLC cell lines. (**D** and **E**) The expression of BRD4 in 95-D and A549 cells is interfered by shRNA and western blotting and flow cytometry confirm the knockdown efficiency.

Then, we further investigated the expression of BRD4 in several NSCLC cell lines, including 95-C, H460, A549 and 95-D cells, which possess different invasion and metastasis potentials by qRT-PCR, western blotting and flow cytometry techniques. As shown in Figure 2A, BRD4 expression in 95-D and A549 cell lines, which have been reported to have higher invasion and metastasis potentials, is much higher than that in 95-C, H460 cell lines (Figure 2B and 2C).

In order to reveal the roles of BRD4 in NSCLC, we designed two short hairpin RNAs (vshRNAs) to suppress the BRD4 expression in 95-D and A549 cells. Our data show that the shRNAs efficiently reduce BRD4 expression (Figure 2D and 2E).

### BRD4 is involved in NSCLC cell invasion, proliferation and apoptosis

**Figure 3:**
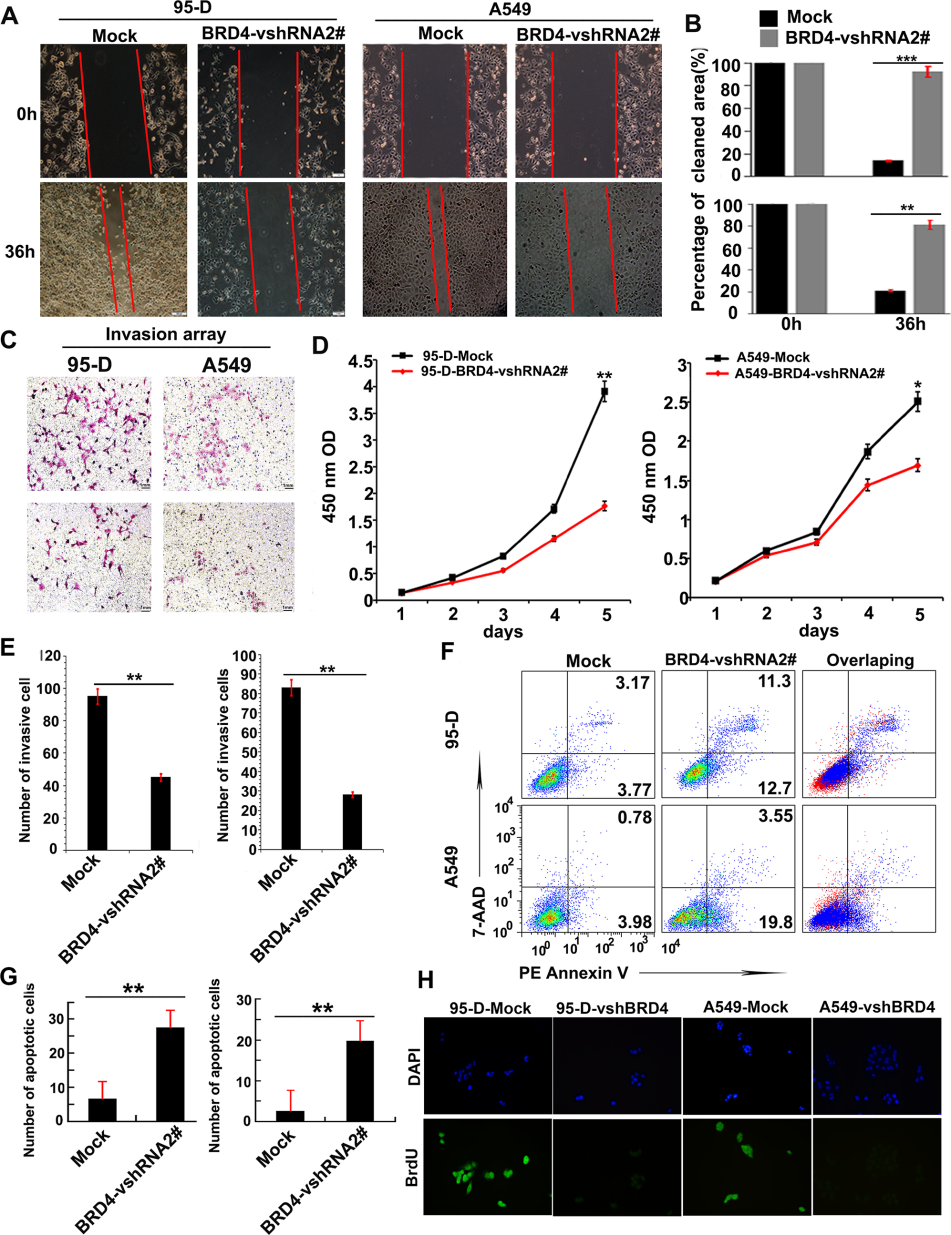
BRD4 is involved in tumor cell invasion, apoptosis and proliferation (**A** + **B**) The mobility ability of NSCLC cells with different BRD4 expressions was determined by the scratch tests. (**C** and **E**) Transwell matrigel invasion analysis detected the number of invasive cells cultured for 24 hours in Transwell chamber 48 hours after transfecting with BRD4-vshRNA. (**D**) Cell counting kit-8 (CCK-8) detects cell proliferation after transfecting with BRD4-vshRNA. (**F** and **G**) FACS detects cell apoptosis using 7-AAD and PE Annexin V staining 48 hours after transfecting with BRD4-vshRNA. (**H**) The cell proliferation was further determined by BrdU.

BRD4 has been shown to combine with Twist to induce epithelial-mesenchymal transition (EMT) in breast cancer in a previous study [18], which may indicate that BRD4 could promote NSCLC cell invasion. So we firstly investigated whether BRD4 may affect the mobility ability and invasion of NSCLC cells. We performed a scratch test, and found that knockdown of BRD4 dramatically inhibited cell mobility ability of A549 and 95-D cells (Figure 3A and 3B). Consistently, the transwell matrigel invasion assay showed that decreasing in the expression of BRD4 by shRNA also impaired invasion of 95-D- and A549 cells (Figure 3C and 3E). Our data indicated that BRD4 could enhance the mobility and invasion ability of NSCLC cells.

Next we determined whether BRD4 played roles in proliferation and apoptosis of NSCLC cells. We measured cell proliferation by a CCK-8 Kit in 95-D- and A549 with or without BRD4 RNA interference. BRD4 vshRNA significantly inhibited the cell proliferation in both 95-D- and A549 cells (Figure 3D), which in line with the BrdU assay (Figure 3H). These results indicated that BRD4 promoted cell proliferation of NSCLC cell lines.

In apoptosis array, 95-D- and A549-BRD4 vshRNA cells presented 27.68 ± 5.55% and 19.73 ± 3.19% cell apoptosis, respectively, which were significantly higher than their corresponding Mock cells (induced only 6.75 ± 2.40% and 2.58 ± 2.20% cell apoptosis, respectively) (Figure 3F and 3G). This indicated that the suppression of BRD4 also affected NSCLC cell apoptosis.

### The BRD4 level was significantly related to histological type, lymph node metastasis, tumor stage and differentiation

Here, we further analyzed the clinicopathological features of NSCLC patients in this cohort of TMAs. 208 cases of primary NSCLC were involved in this analysis, the cohort contained 148 males and 60 females, and 85 squamous cell carcinomas, 110 adenocarcinomas and 13 other pathologic subtypes of NSCLC (including adenosquamous carcinoma, large-cell carcinoma, mucoepidermoid carcinoma and carcinosarcoma). Moreover, 144 tumors were in TNM stages I–II and 64 tumors were in stages III–IV. In addition, 93 tumors were low differentiation, and 115 tumors were highly differentiated. The statistical details about these patients and the relationship between BRD4 levels and the clinicopathological features were exhibited in Table 1.

**Table 1:** Correlation between BRD4 and clinicopathological characteristics in 208 NSCLCs

Variables	No. of patients	BRD4 expression level		
		low	high	*P*
**Age**				
< 60	102	61	41	0.097
≥ 60	106	51	55	
**Gender**				
Male	148	71	77	0.009
Female	60	41	19	
**Smoking status**				
Smokers	84	43	41	0.572
Non-smokers	124	69	55	
**Histological type**				
Squamous cell carcinoma	85	37	48	*0.006
Adenocarcinomas	110	70	40	
Other^a^	13	5	8	
**Tumor stage**				
I–II	144	97	47	< **0.001**
III–IV	64	15	49	
**Lymph node metastasis**				
Yes	90	27	63	< **0.001**
No	118	85	33	
**Tumor size**				
< 3 cm	69	43	26	0.104
≥ 3 cm	139	69	70	
**Differentiation**				
Well/moderate	115	74	41	**0.0031**
Poor	93	38	55	

NOTE: Bold values are statistically significant (*P* < 0.05).

a Other including adenosquamous carcinoma, large-cell carcinoma, mucoepidermoid carcinoma and carcinosarcoma.

* *P* value was analyzed by squamous cell carcinomas vs. adenocarcinomas.

**Figure 4:**
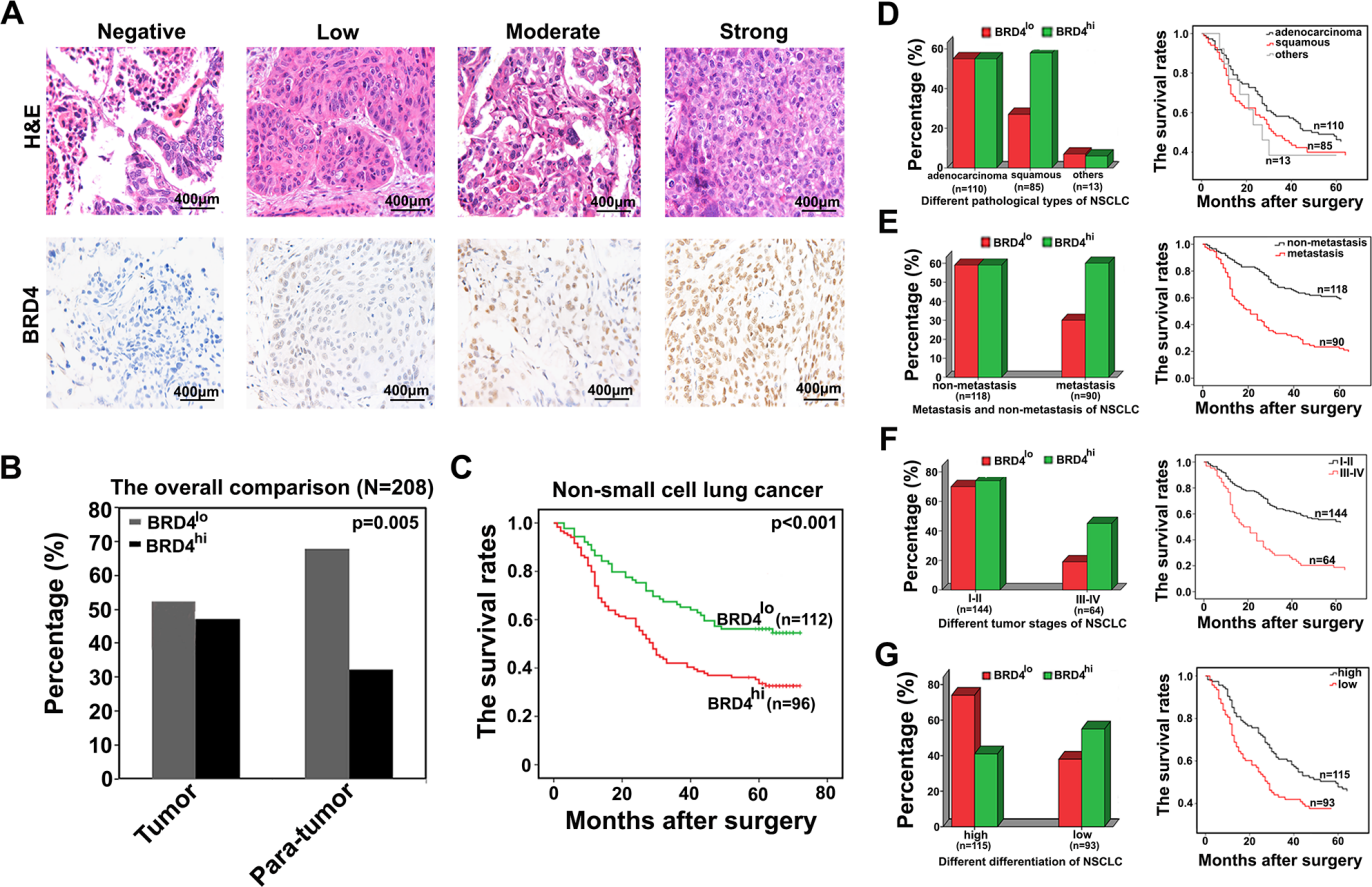
High level of BRD4 is associated with the poor prognosis of NSCLC patients (**A**) Representative pictures of immunostaining show that BRD4 levels in NSCLC tissues vary greatly. (**B**) The proportion of BRD4^hi^ expression in NSCLC tissues and their corresponding adjacent normal lung tissues is 46.15% and 32.21%, respectively. (**C**) The overall survival of patients with differential BRD4 expression levels. (**D–G**) The subgroup analysis of the relationship between BRD4 level and different clinicopathological characteristics of NSCLC patients as well as the evaluation of their corresponding overall survival was performed here.

The BRD4 levels in NSCLC tissues varies greatly (Figure 4A), and we dichotomized them into BRD4^hi^ level (moderate and strong; *n =* 96) and BRD4^lo^ level (negative and weak; *n =* 112) groups. Then we analyzed the relationship between BRD4 levels and the clinicopathological parameters of NSCLC. We observed that BRD4 level was significantly related to histological type (*P* < 0.001), lymph node metastasis (*P* < 0.001), tumor stage (*P* < 0.0031) and differentiation (*P =* 0.031). However, other clinicopathological features, including age, smoking status, were not directly associated with the BRD4 level.

We further compared the proportion of BRD4 expression between tumor and para-tumor tissues, we found that BRD4^hi^ level in NSCLC tissues accounted for 46.15% (96 of 208), which was higher than that in para-tumoral tissues (32.21%, 67 of 208) (*p =* 0.005, Figure 4B).

### High BRD4 level correlated to poor prognosis of NSCLC patients

At the end of following-up, 120 patients died from the recurrence or metastasis of disease. The 5-year overall survival rate after surgery for all patients was 47.6%. Statistically, there was a strikingly inverse association between BRD4 intensity of immunostaining and OS (*p* < 0.001, Figure 4C). The median OS time for BRD4^hi^ level patients was 34.18 months, compared with 45.93 months for BRD4^lo^ level patients. Multivariate analysis revealed that BRD4 expression level in cancer tissues was an independent prognosticator for OS (Table 2). Therefore, BRD4 is a valuable predictor of OS for NSCLC patients.

**Table 2:** Univariate and multivariate analysis of factors associated with OS

Variables	Univariate analysis			Multivariate analysis		
	HR	95% CI	*P*	HR	95% CI	*P*
**Gender**						
(male *vs.* female)	0.789	0.526–1.183	0.251			
**Smoking status**						
(non-smokers *vs.* smokers)	1.284	0.895–1.843	0.175			
**Tumor size**						
(≥ 3 cm *vs.* < 3 cm)	0.363	0.232–0.569	< 0.001	0.491	0.309–0.780	0.003
**Lymph node metastasis**						
(no *vs. ye*s)	3.042	2.103–4.399	< 0.001	2.131	1.361–3.337	0.001
**Tumor stage**						
(I–II *vs.* III–IV)	2.771	1.922–3.993	< 0.001	1.521	0.982–2.356	0.060
**Differentiation**						
(well/moderate *vs.* poor)	1.431	1.000–2.049	0.050	1.158	0.802–1.673	0.434
**BRD4 level**						
(low *vs*. high)	1.827	1.274–2.621	0.001	1.457	1.014–2.095	0.042

**Abbreviations and note:** OS, overall survival; 95% CI, 95% confidence interval. Variables were adopted for their prognostic significance by univariate analysis with forward stepwise selection (forward, likelihood ratio). Variables were adopted for their prognostic significance by univariate analysis (*P* < 0.05).

We also found that BRD4 levels were different among NSCLC patients between squamous cell carcinomas and adenocarcinomas, between lymph node metastasis and lymph node non-metastasis, between TNM stages I–II and TNM stages III–IV and between low differentiation and high differentiation, we performed a subgroup analysis classified by these clinicopathological features. We observed that high level of BRD4 more frequently appeared in patients with squamous cell carcinomas, with lymph node metastasis, with TNM stages III–IV and with low differentiation. Clinically, It is certain known that the patients associated with high level of BRD4 are more inclined to short OS (Figure 4D–4G). These results further confirmed the perspective that BRD4 may be a valuable prognosticator of NSCLC patients.

## DISCUSSION

Bromodomains (BRDs) are epigenetic readers that selectively recognize acetylated lysine residues on histone protein tails, and are the only known protein molecules that target lysine residues [19]. In recent years, bromodomain containing protein 4 (BRD4) has become the research hotspot of biomedical fields. Although recent elucidation of the diagnostic, prognostic and therapeutic value of BRD4 in lots of cancers, such as breast cancer, acute myeloid leukemia, and colorectal cancer [20–22], its role in non-small cell lung cancer (NSCLC) remains elusive. Here, we found BRD4 expression was significantly upregulated in NSCLC tissues compared to their corresponding peritumoral normal lung tissues. Moreover, the suppression of BRD4 expression can impair cell invasion, inhibit cell proliferation and accelerate cell apoptosis. More importantly, high level of BRD4 expression closely correlated to the poor prognosis of NSCLC patients. Thus, we may present such a prospective that NSCLC cells with BRD4^hi^ level could protect themselves by enhancing their proliferation and invasion abilities, which may finally cause an unfavorable prognosis for NSCLC patients.

Moreover, a credible result identified in this study is the relationship between high BRD4 expression and poor prognosis of NSCLC patients. This finding may be supported by the fact that aggressive histopathological characteristics, such as lymph node metastasis, poor differentiation, and high TNM staging of NSCLC were significantly more frequent in patients with BRD4^hi^ level than in those with BRD4^lo^ level. This indicates that BRD4 protein expression may be a powerful prognostic indicator for NSCLC. BRD4, as a member of the bromodomain and extra terminal domain (BET) family of bromodomain-containing proteins, is also a proto-oncogene that can be mutated via chromosomal translocation in a rare form of squamous-cell carcinoma [23], although the role in NSCLC has not been described. The recent research development about BRD4 [24, 25], together with our screening results, prompted us to further investigate the suitability of BRD4 as an NSCLC therapeutic target.

Summarily, our data show that BRD4 is a novel marker in predicting the prognosis of NSCLC patients, and a potential therapeutic target for NSCLC patients.

## MATERIALS AND METHODS

### Patients and specimens

All specimens were achieved from 208 NSCLC patients who received curative resection at Zhongshan Hospital of Fudan University (Shanghai, People's Republic of China) in 2005. The collection and conservation of samples and details of the cohort are in agreement with the description of our previous study [27]. Briefly, tumor stage was adjudged on the basis of the tumor-node-metastasis (TNM) 7th edition of International Union Against Cancer Staging Manual. Pathological classification was ruled according to the World Health Organization criteria. Following-up was terminated in July 2010. The median follow-up was 43 months (range from 1 to 66 months). Overall survival (OS) was defined as the interval between surgery and death or between surgery and the last observation for surviving patients. Twelve fresh NSCLC and their adjacent normal lung samples were obtained from 208 consecutive patients. Ethical approval was obtained from the Zhongshan Hospital Research Ethics Committee, and written consents were obtained from all patients.

### Immunohistochemistry and tissue microarrays

The immunostaining and tissue microarrays (TMAs) were performed as previous study [27]. Immunohistochemistry (IHC) staining for the target genes was carried out on sections of the formalin-fixed samples on the TMAs.

### Cell culture

95-C, H460, A549 and 95-D cell lines were purchased from the Institute of the Biochemistry and Cell Biology of Chinese Academy of Science. A549 and H460 cell lines were grown in Dulbecco's modified Eagle's medium (DMEM) (Gibco) supplemented with 10% fetal bovine serum (FBS), 2 M glutamine, 100 IU/ml penicillin, and 100 μg/ml streptomycin sulfate. 95-C and 95-D were grown in 1640 (Gibco) supplemented with 10% FBS, 2 M glutamine, 100 IU/ml penicillin, and 100 ug/ml streptomycin sulfate. All cell lines were incubated in atmosphere of 5% carbon dioxide at 37 degree.

### Western blotting

Thirty micrograms of total cell extract protein was resolved by SDS-polyacrylamide gel electrophoresis, transferred onto polyvinylidene difluoride membranes, and incubated with the corresponding primary antibodies. The membranes were developed with the enhanced chemiluminescence method (Pierce, Rockford, IL, USA). The primary antibody used in this study was BRD4 (1:1000 dilutions, Serotec). β-actin (1:5000; Chemicon, USA) was used as an internal control. All experiments were performed in triplicate.

### RNA extraction and real-time polymerase chain reaction

Total RNA of cultured cells was extracted with TRIzol reagent (Invitrogen, Carlsbad, CA, USA) according to the manufacturer's protocol. Amplification and detection were performed using the ABI PRISM 7500 Sequence Detection System (Applied Biosystems, Foster City, CA, USA) starting with 1 ul cDNA and SYBR Green Real time PCR Master Mix (Toyobo, Japan). β-actin was used as an internal control. Primer sequences of BRD4 and β-actin were listed in Table 3. The relative expression of BRD4 messenger RNA (mRNA) was analyzed by the comparative cycle threshold (Ct) method. All experiments were performed in triplicate.

**Table 3:** Primer and vshRNA sequences used in this paper

**BRD4 sequences**	
Forward	5′TTTGAGACCCTGAAGCCGTC′3
Reverse	5′TAGGCAGGACCTGTTTCGGA′3
**β-actin sequences**	
Forward	5′ACCAACTGGGACGACATGGA′3
Reverse	5′CCCTCGTAGATGGGCACAGT′3
**BRD4 vshRNA1#**	
Sense sequence	5′CCGGCAGTGACAGTTCGACTGATGACTCGAGTCATCAGTCGAACTGTCACTGTTTTTTG′3
Anti-sense sequence	5′AATTCAAAAACAGTGACAGTTCGACTGATGACTCGAGTCATCAGTCGAACTGTCACTGT′3
**BRD4 vshRNA2#**	
Sense sequence	5′CCGGCCTGGAGATGACATAGTCTTACTCGAGTAAGACTATGTCATCTCCAGGTTTTTG′3
Anti-sense sequence	5′AATTCAAAAACCTGGAGATGACATAGTCTTACTCGAG TAAGACTATGTCATCTCCAGG′3

### Gene silencing and transfection

Two different sequences targeted to two different sites in BRD4 mRNA were designed and provided by Ribobio (Guangzhou, Guangdong, China). The sense and antisense strands of short hairpin RNA (shRNA) are shown in Table 3. Transfection was performed in cells using Lipofectamine 2000 (Invitrogen, USA).

### Cell proliferation and invasion assay

Cell proliferation was measured by a CCK-8 Kit (Dojindo Laboratories, Rockville, MD). The procedures were performed strictly in accordance with the instruction provided by the kit. Cell invasion analysis was performed using a Transwell (Corning, NY) as described in the reference [28]. All assays were performed in triplicate.

### Flow cytometry analyzes cell apoptosis

Flow cytometric (FCM) analysis was performed to inspect cell apoptosis. The proportion of cells undergoing apoptosis was evaluated using PE Annexin V Apoptosis Detection Kit I (BD Pharmingen^™^, Cat: 559763). Cells were discriminated into three populations, viable cells, early apoptotic cells and late apoptotic or necrotic cells based on PE Annexin V and 7-AAD staining. Apoptosis array was analyzed using FACSCarlibur (BD Pharmingen^™^). All assays were repeated for three times.

### Evaluation of immunostaining intensity of TMAs

The BRD4 was immunohistochemically stained yellow or brown positioning in the cell nucleus. The samples were scored independently according to the intensity of cellular staining and the proportion of stained cells [27]. The proportion of stained cells in total cell number of every point about 0, 1% to 25%, 25% to 50%, 50% to 75%, > 75% were scored as 0, 1, 2, 3, 4 points, respectively. The intensity of cellular staining was determined by the degree of color, namely none, weak (yellow), medium (brown), and strong (Brown Brown) were scored as 0, 1, 2, 3 points, respectively. The final combined score about 0–1, 2–3, 4–5 and 6–7 points were divided into negative(−), weak positive (+), moderate positive (+ +) and strong positive (+ + +), respectively. Then we defined the negative and weak positive staining as the BRD4^lo^ level group, and the moderate and strong positive staining as the BRD4^hi^ level group, respectively.

### Statistical analysis

Statistical analyses were performed with SPSS 17.0 software (SPSS, Chicago, IL) and PRISM 5.0 (GraphPad Software Inc., San Diego, CA, USA) as previously described [29]. Overall survival (OS) was calculated using the Kaplan-Meier method and was analyzed using the log-rank test. A Cox proportional hazards regression model was used to analyze independent prognostic factors. For comparisons of individual variables, *t*-tests, chi-square tests, Fisher exact tests, and Spearman coefficient tests were used when necessary. All *P*-values obtained were considered as significant when ≤ 0.05.
